# Changes in Richness, Abundance, and Occurrence of Beetles in South Korea over Ten Years: Identifier Bias and Selection of Climate Change Indicators

**DOI:** 10.3390/insects17020156

**Published:** 2026-01-30

**Authors:** Tae-Sung Kwon, Sung-Soo Kim, Go-Eun Park, Youngwoo Nam

**Affiliations:** 1 Alpha Insect Diversity Lab, Nowon, Seoul 01746, Republic of Korea; insectcom@naver.com; 2 Research Institute for East Asian Environment and Biology, Amsa-3 dong, Gangdong-gu, Seoul 05236, Republic of Korea; nabifri@naver.com; 3 Forest Ecology Division, National Institute of Forest Science, Seoul 02455, Republic of Korea; goeunpark@korea.kr; 4 Forest Entomology and Pathology Division, National Institute of Forest Science, Seoul 02455, Republic of Korea

**Keywords:** Coleoptera, climate warming, abundance, distribution, diversity, Ecological Temperature Index

## Abstract

Climate change is rapidly altering the distribution and abundance of species, with significant impacts on regional ecosystems, including reduced ecosystem services and the loss of biodiversity. Accurately predicting changes in the distribution and abundance of taxa under future climate scenarios is, therefore, crucial. In South Korea, beetle data collected via pitfall traps from approximately 300 forest sites between 2007 and 2009 (30 families, 4 genera, and 150 species) were used to forecast changes in their abundance and distribution under climate change scenarios RCP 4.5 and 8.5. This study evaluated the accuracy of those predictions using data from a subsequent survey conducted between 2017 and 2019. We compared species richness, abundance, changes in abundance (i.e., number of individuals), and occurrence (i.e., number of occupied sites) using data from 273 sites that were surveyed in both the initial (2007–2009) and follow-up (2017–2019) periods. All four parameters were found to be significantly influenced by the identifiers. This identifier bias was attributed to the omission of morphologically similar species in the initial survey or the loss of individuals during the preparation process of dry specimens. As a result, increases in abundance and distribution appear to have been affected by identification errors, whereas decreases more closely reflect actual ecological changes. When the comparison between predicted and observed results was restricted to taxa with reduced abundance and distribution, the number of taxa that matched the predictions was significantly higher than that of those that did not. Based on ease of identification, abundance, and sensitivity to climate change, we selected a set of indicator taxa (four families, two genera, and seven species) for climate change monitoring.

## Simple Summary

Climate change has significant effects on insect populations, yet long-term monitoring data are limited. As part of this study, we examined changes in beetle richness, abundance, and occurrence across 273 forest sites in South Korea, comparing data collected a decade apart (2007–2009 vs. 2017–2019). We found significant underestimation of abundance of beetles during the species identification process in the first survey. After accounting for this bias, we compared changes observed with earlier predictions based on species distribution models and ecological temperature indices. Notably, taxa that declined in abundance and occurrence matched the predicted trends more accurately than those that did not, suggesting real ecological changes, likely driven by climate warming. Based on abundance, ease of identification, and temperature sensitivity, we propose four beetle families, two genera, and seven species as effective indicators for long-term climate change monitoring in forest ecosystems.

## 1. Introduction

Beetles represent one of the most diverse groups of organisms on Earth [1,2]. They first appeared during the Carboniferous period [3] and account for approximately 20% of all known animal and plant species [1]. Beetles occupy nearly all terrestrial habitats, except for polar regions, and play a crucial role in maintaining ecosystem functions through diverse services [2]. In the Permian period, wood-feeding beetles were dominant and played a key role in the global carbon cycle by decomposing dead plant material [3]. Even today, various beetle families, such as Cerambycidae, Buprestidae, and Scolytidae, continue to decompose dead wood, facilitating carbon cycling [2]. In the African savannah, vast populations of large herbivorous mammals generate enormous quantities of dung, which are rapidly processed by a diverse assemblage of dung beetles [4]. Similarly, in temperate deciduous forests, the leaf litter layer remains relatively constant due to the rapid decomposition of autumnal leaves, driven by numerous micro-beetles in collaboration with microorganisms and microfauna [5]. Beetles also fulfill critical ecological roles as pollinators, predators, herbivores, and prey for birds and mammals [1,2,5]. Despite these beneficial functions, certain pest species (e.g., *Monochamus alternatus*, *Tomicus piniperda*, and *Agelastica coerulea*) within Cerambycidae, Scolytidae, and Chrysomelidae cause significant ecological and economic damage [6].

Climate-driven changes in beetle distribution are expected to have profound consequences on species composition and ecosystem functions. Currently, species distribution models (SDMs) are widely used to study changes in species ranges under climate change scenarios [7,8,9]. However, research on beetle distribution under climate change has primarily focused on economically damaging pest species [10,11,12], whereas studies on non-pest beetles remain scarce despite their vital ecological roles [13,14]. The few studies focusing on these species are limited to a single species or taxon, such as scarab beetles (family Scarabaeidae), rather than encompassing the full diversity of the order Coleoptera. Given their immense diversity and critical ecological functions, predicting future changes in the abundance and distribution of common, non-pest beetles is essential for understanding how terrestrial ecosystems will respond to climate change. An exception to this gap is found in South Korea, where studies predicted the future abundance and distribution of beetles (30 families, 4 genera, and 150 species) for the period 2056–2065 under RCP 4.5 and 8.5 climate scenarios [15,16,17]. This study utilized data collected from pitfall traps at approximately 300 forest sites between 2007 and 2009 [15]. To assess long-term changes, beetle surveys were repeated at the same sites, a decade later, from 2017 to 2019. Recognizing the importance of these findings, the National Institute of Forest Science incorporated beetle surveys into a long-term climate change monitoring program, conducting a third round of surveys between 2022 and 2024, with plans to continue monitoring every five years [18].

In this study, we evaluated predictions of species distribution using beetle survey data collected during the second survey period (2017–2019). The predictions were made for the period 2056–2065, approximately 30 years into the future. However, considering that global temperatures are already rising, it was assumed that the predicted trends (i.e., increases or decreases in abundance and occurrence) may already be observable. However, the comparison of beetle fauna indicates serious identifier bias rather than genuine ecological change. Therefore, we analyzed the effects of identifier bias on species richness, abundance, occurrence, and their change. Our analysis provides clear evidence of identifier bias; evaluations of prediction accuracy were conducted after controlling for this bias. Based on the results, we identified several taxa as potential indicators for the long-term monitoring of climate change impacts.

## 2. Materials and Methods

### 2.1. Sites and Survey

Vegetation in South Korea is classified as temperate deciduous forest [19,20], which covers approximately 65% of the national land area [21]. Naturally occurring forests are primarily composed of oak and pine species, and extensive plantations, including Korean pine (*Pinus koraiensis*), Japanese larch (*Larix kaempferi*), and Pitch pine (*Pinus rigida*), are also present [22]. Historically, pine forests were widespread; however, their extent has steadily declined due to pine wood nematode infestation, global warming, forest fires, and the succession of natural vegetation. In contrast, oak forests have gradually expanded in recent decades [22].

The national mean annual temperature between 1960 and 2010 was 13.4 °C, increasing by 0.025 °C per year [23]. In contrast, the mean annual temperature at the survey sites in this study was 10.32 ± 1.72 °C (SD), ranging from 5.73 to 13.78 °C (Table S1). This discrepancy likely reflects the location of most meteorological observation stations in low-lying urban areas; by contrast, our survey sites are located at relatively higher elevations and farther from urban centers, minimizing the effects of urban heat islands. During the survey period (2007–2017), the national mean annual temperature was 13.3 °C, and the average annual precipitation was 1318.9 mm (Table S1). No significant change was detected in either temperature or precipitation between 2007 and 2019.

**Figure 1:**
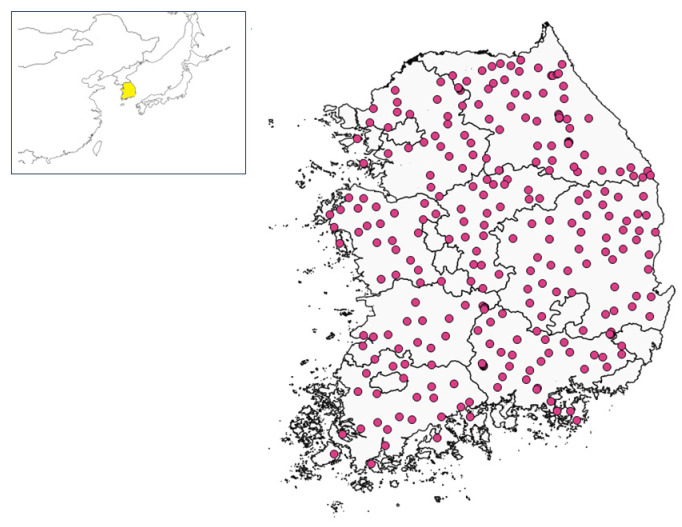
Study sites (n = 273, red points) for beetle monitoring in South Korea.

This study was conducted at 273 inland forest sites in South Korea (Figure 1, Table S2). During the first survey (2007–2009), data were collected from 314 sites. During the second survey a decade later (2017–2019), 293 sites were surveyed (Table S2). Of these, 273 sites were identified to be common to both periods and were used to analyze temporal changes in beetle communities (Table S2). These common survey sites were distributed across inland South Korea and spanned the following elevations: 169 sites were located below 300 m; 57 sites were located between 300 and 600 m; 28 sites were located between 600 and 1000 m; and 19 sites were situated in highland areas above 1000 m (Table S2). The 293 sites from the second survey were designated climate change arthropod monitoring sites by the National Institute of Forest Science, with surveys being conducted at five-year intervals [18]. The third survey was conducted during the 2022–2024 period. In terms of vegetation, surveyed sites included 142 oak forests, 65 red pine forests, 26 larch forests, and 40 sites classified as other forest types (Table S2).

The arthropod survey was conducted from May to September, which is a period of peak activity. The study was carried out over three years, surveying approximately 100 sites per year. In the first year, surveys were conducted in the central regions of Gyeonggi-do and Gangwon-do; in the second year, surveys were conducted in the southeastern regions of Gyeongsangbuk-do and Gyeongsangnam-do; and in the third year, surveys were conducted in the southwestern regions of Chungcheongbuk-do, Chungcheongnam-do, Jeollabuk-do, and Jeollanam-do. Each site was surveyed once. To minimize the influence of seasonal variation, the survey periods were matched as closely as possible between the first and second survey cycles. Beetles were collected using pitfall traps, consisting of white plastic containers (95 mm in diameter and 60 mm in height). Ten traps were installed at 5 m intervals, flush with the ground surface, and retrieved after 10–15 days. Each trap was filled to approximately one-third of its volume with automotive antifreeze (ethylene glycol) as a preservative. Upon retrieval, the preservation fluid was removed using a fine iron mesh screen. The contents of all 10 traps from a site were combined into a single plastic container and preserved in 95% ethyl alcohol.

### 2.2. Reported Projection for Abundance and Occurrence of Beetles in South Korea

Based on the RCP 4.5 and RCP 8.5 climate change scenarios [24], 18 common beetle species in South Korea (occurrence frequency ≥ 10%) with abundances strongly correlated with temperature were selected for future projections (2056–2095) [15]. At the time of modeling, RCP 8.5 was the standard climate change scenario used in South Korea. For these projections, the national temperature range was divided into five intervals (3–7 °C, 7–9 °C, 9–11 °C, 11–13 °C, and 13–15 °C). The mean abundance of each species within these intervals, derived from the first survey period, was used to estimate future abundance based on projected temperatures. A regression equation was then established between predicted changes in national mean abundance and each species’ Ecological Temperature Index (ETI: the mean annual temperature of the sites where the species occurred). This relationship was used to qualitatively predict whether abundance would increase or decrease for an additional 108 species that were either slightly common (occurrence frequency 1–10%) or common (≥10%) but were not included in the original prediction. The same method was also applied at the family level, enabling quantitative projections for 8 families and qualitative predictions for 23 families [16]. Additionally, using a Generalized Additive Model (GAM) with annual mean temperature and precipitation as explanatory factors, we projected future changes in the abundance of 14 common species. The species selected were strongly correlated with these climate variables and existed under similar climate scenarios [17]. Finally, the predicted changes in abundance (increase or decrease) from these models or using ETI were compared with the changes observed in this study; positive values indicated increases, while negative values indicated decreases.

### 2.3. Identification

Beetle identification was conducted by various personnel in the first and second surveys (Table 1). Beetles were separated from arthropod samples collected via pitfall traps and preserved in 95% ethyl alcohol. After separation, individuals were counted at the order level (i.e., total beetle abundance per sample; hereafter, sample abundance) and stored in separate plastic containers containing 95% ethyl alcohol. The identification of specimens was performed by trained laypersons: Female A during the first survey and Female B during the second. Dried specimen preparation was carried out by A in the first survey and by T.-S. Kwon for the second survey. In the first survey, because A lacked prior experience in beetle identification, the working protocol required all beetles to be mounted as dried specimens. However, for species collected in large numbers, only a subset of individuals was dried; the remaining individuals were counted and recorded by species. By contrast, the second survey was conducted by T.-S. Kwon, who had experience in beetle identification. For each species, 1–3 representative individuals were prepared as dried specimens, and all individuals were counted. For commonly occurring species, identification was based on reference specimens, whereas all rare species were mounted individually as dried specimens. Species identification was conducted twice using dried specimens, as described below.

In the first survey, Carabidae specimens were identified by C.M. Lee, an ecologist with expertise in Japanese carabid beetles, and the remaining beetle taxa were identified by S.-S. Kim, who is a specialist in moth taxonomy using the published taxonomic literature [25,26,27]. Subsequently, all initially identified specimens were re-examined by beetle specialists. Carabidae were re-identified by J.K. Jung, a Korean carabid ecologist; Staphylinidae were re-identified by S.K. Lee, a rove beetle (Staphylinidae) taxonomist; and the remaining families were re-identified by S.W. Park, a Curculionidae expert. S.W. Park, who has extensive experience in identifying a broad range of beetle taxa, received additional assistance from other specialists [28] for certain groups, such as Elateridae, Cerambycidae, Nitidulidae, and Melolonthidae. During this re-identification process, the experts also examined a large number of beetle specimens stored at the National Institute of Forest Science, which were collected at numerous forest sites since the early 2000s. Approximately 1400 re-identified species were curated as reference specimens in the Insect Specimen Depository of the National Institute of Forest Science, and photographs of these specimens were published in two reference volumes [29,30]. The comparison of initial and re-identifications showed that the overall concordance rate was only 21% with instances of both splitting a single species into multiple taxa and combining multiple species into one [30].

In the second survey, beetle identification was carried out by T.-S. Kwon. The initial identification was performed using the published literature [25,26,27,29,30], and was subsequently revised by comparing with reference specimens. The body length for each species was measured from dried specimens using a vernier caliper; the representative body length of a species was defined as either a medium-sized individual or the median value of the smallest and largest individuals (Table S3).

The Ecological Temperature Index (ETI) was calculated for 87 taxa used in the analyses of abundance and occurrence changes. ETI was computed using the following equation:

ETI = ∑(Ai × Ti)/∑Ai,

where Ai is the abundance (number of individuals) at site i and Ti is the annual mean temperature at site i. ETI was calculated separately for the first and second surveys, and the median of the two values was used as the final ETI for each taxon. The method used to estimate the annual mean temperature for each survey site is described in Kwon et al., 2023 [17]. ETI is a generalized term of the Species Temperature Index (STI), originally proposed in Devictor et al., 2008 [31], and has been extended in this study to apply at both the genus and family levels.

### 2.4. Data Analysis

The effects of various identifiers on beetle abundance, species richness, and changes in abundance and occurrence were analyzed using the following process. To assess the relationship between the number of individuals counted at the order level (sample abundance) and those identified at the species level (identification abundance) in both surveys, regression analyses were performed. In these models, the identification of abundance was set as the dependent variable, and sample abundance was set as the independent variable. A higher coefficient of determination (*R*^2^) and a regression slope closer to one indicated a smaller number of missed individuals during the species-level identification process.

Species richness (number of species) between the surveys was compared using the specaccum function from the vegan package in R 4.1.1 [32]. The mean and standard deviation were calculated when the number of sampling sites reached 272. Using these values, richness in the first and second surveys was compared using Welch’s *t*-test [33]. The second survey had more than twice the richness of the first survey (*p* << 0.001). This dramatic increase is unlikely to reflect a natural ecological phenomenon and more likely to reflect losses during the identification in the first survey, particularly among rare or low-abundance species. To assess whether identifier bias contributed to species loss, species richness was compared specifically for the families Carabidae, Staphylinidae, and Cerambycidae. Carabidae specimens were identified by a Japanese Carabidae expert (C.M. Lee) in the first survey; however, T.-S. Kwon performed the identification for the second survey, with relatively less experience in Carabidae identification. Staphylinidae are known for their morphological similarity among species and a high number of cryptic micro-species [2,27]; as such, larger discrepancies were expected due to the identifier effect, and Cerambycidae, with a large body size and distinctive morphological features, were expected to show smaller differences between identifiers.

We tested whether the difficulty of taxonomic identification influenced changes in abundance (number of individuals) and occurrence (number of occupied sites). The analysis included 24 families, 33 genera, and 30 species that were present in both surveys and occurred at more than 10% of both survey sites (*n* > 27) (Table S4). The difficulty of identification was classified as “easy” or “difficult” based on T.-S. Kwon’s expert assessment. We tested whether changes in abundance and occurrence differed between these two groups. Changes in abundance were calculated as the average difference in log-transformed abundance values:

ln(N_2_ + 1) − ln(N_1_ + 1),

where N_1_ and N_2_ represent the abundance in the first and second surveys, respectively. Survey sites where the taxon was absent in both surveys were excluded from the calculation. Changes in occurrence for each taxon were calculated as the difference between log-transformed values for the number of occupied sites:

ln(O_2_) − ln(O_1_),

where O_1_ and O_2_ denote the number of sites where the taxon occurred in the first and second surveys, respectively. The nonparametric Wilcoxon Rank-Sum test was used to assess whether the changes in abundance and occurrence differed significantly between the easy and difficult-to-identify groups.

**Figure 3:**
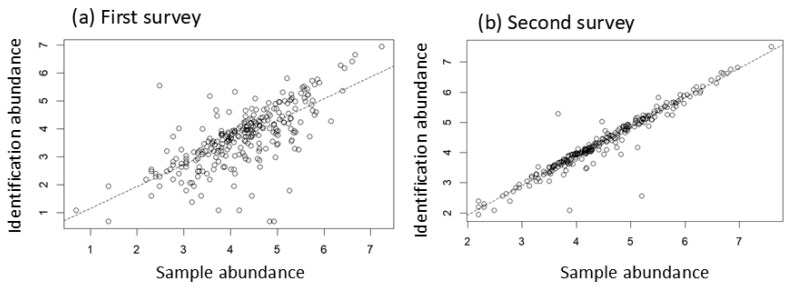
Abundance of beetles from the first (2007–2009) and second (2017–2019) surveys in South Korea. Abundance (number of individuals) is log-transformed (ln(n + 1)). Sample abundance is the number of beetles used for arthropod identification (order level) in the pitfall traps, and identification abundance is the number of beetles used for beetle identification (species level). Sample abundance and identification abundance in the first survey (**a**), and the second survey (**b**). The dashed lines are the significant (*p* < 0.05) regression models. In regression, (**a**) Y = 0.37 + 0.78 × X, adj. *R*^2^ = 0.51, *F*_1,271_ = 283.1, *p* << 0.001, and (**b**) Y = −0.01 + 0.97 × X, adj. *R*^2^ = 0.93, *F*_1,271_ = 3458, *p* << 0.001.

The prediction results from the previous studies [15,16,17] were compared with the observed data from this study using a chi-square (*χ*^2^) test. Because identifier effects primarily underestimated abundance in the first survey (Figure 3), observed increases in abundance and occurrence may reflect identifier bias, whereas observed decreases are more likely to represent actual ecological changes. Accordingly, analyses focused on taxa showing decreases in abundance and occurrence. For these taxa, the numbers of matching versus non-matching predictions were compared using the χ^2^ test. All statistical analyses were conducted using R [34].

## 3. Results

**Figure 2:**
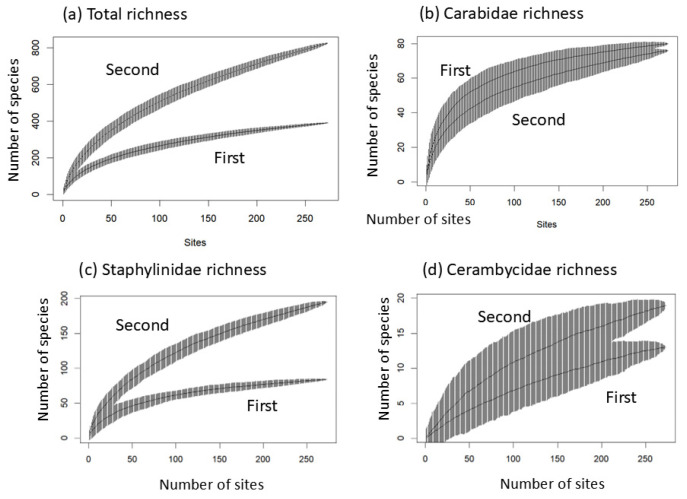
Curves of beetle species accumulation across 273 sites. Mean and standard deviation of richness are randomly permuted using specaccum function from vegan package in R. First: first survey in 2007–2008; second: second survey in 2017–2019.

A total of 53,788 beetles representing 69 families and 1161 species were collected across the two surveys (Tables S5 and S6). Only 149 species (12.8% of the total) were found in both surveys. The first survey recorded 48 families and 388 species, whereas the second survey recorded 65 families and 822 species, with substantially higher diversity (Table S6). Species richness was estimated at 272 sites using permutation methods, yielding 389.57 ± 0.71 species (mean ± SD) in the first survey and 823.34 ± 1.62 in the second, i.e., more than a twofold increase (*t* = −4045.9, *p* << 0.0001). However, comparisons between three beetle families with differing identification characteristics revealed effects based on the identifier. For Carabidae, identified by a carabid expert (C.M. Lee) in the first survey, species richness was 79.95 ± 0.22, compared to 75.96 ± 0.20 in the second survey (Figure 2). Unlike the other families, species richness was higher in the first survey, highlighting a highly significant difference between the two periods (*t* = 223.2, *p* << 0.0001). Although this decline might partly reflect identifier effects, given that identifier bias was mainly due to undercounting in the first survey (see below), the results also likely reflect a genuine ecological change. The richness of Staphylinidae increased from 83.9 ± 0.33 in the first survey to 194.76 ± 0.61 in the second survey, more than doubling the original amount and mirroring the overall pattern showing an increase in total species richness (Figure 2, *t* = −2656.18, *p* << 0.0001). The richness of Cerambycidae increased from 12.95 ± 0.22 to 18.98 ± 0.14, a smaller yet statistically significant increase (Figure 2, *t* = −337.67, *p* << 0.0001). The influence of the identifier was especially pronounced in Staphylinidae, but even large-bodied, morphologically distinctive taxa such as Cerambycidae appeared to be affected by differences in expertise.

The influence of the identifier is clearly demonstrated by comparing the abundance of samples and identified species (Figure 3). In Figure 3, sample abundance refers to the total number of beetles (order level) extracted from the ten pitfall traps at each site, and identification abundance is the sum of individuals identified at the species level. Comparisons of these two measures reveal substantial differences between the first and second surveys (Figure 3). Ideally, if no beetles were lost during identification, the regression intercept would be zero and the slope would be one. In the second survey, the intercept is close to zero (−0.01 ± 0.08, mean ± SE) and the slope is close to one (0.97 ± 0.02), indicating minimal loss. In contrast, the first survey shows a higher intercept (0.37 ± 0.2) and a lower slope (0.78 ± 0.05), indicating considerable loss during the identification process.

**Figure 4:**
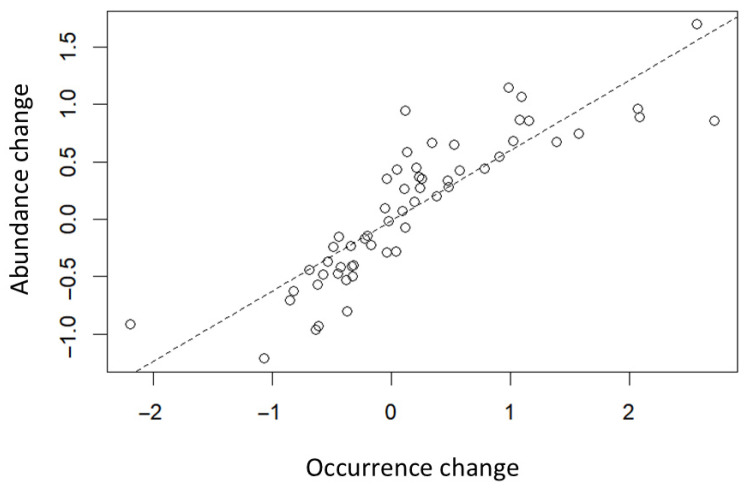
Relationship between changes in occurrence and abundance for 87 taxa. The hatched line indicates the regression model. In the regression model, Y = 0.04183 + 0.47715 × X, adj. *R*^2^ = 0.72, *F*_1,85_ = 225.4, and *p* << 0.001.

**Figure 5:**
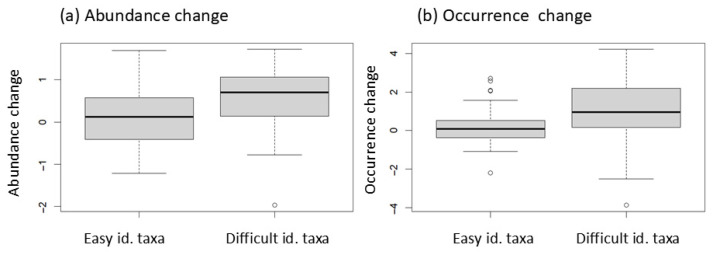
Change in abundance and occurrence of beetles between the first (2007–2009) and second (2017–2019) surveys. The analyzed taxa (family, genus, and species; *n* = 87) occurred most commonly at 10% of sites (28 or more) in the first or second survey. Easy (*n* = 58) and difficult (*n* = 29) identification taxa were determined subjectively by KT-S (Table S4). (**a**) Changes in abundance for each taxon are the mean abundance change (ln(n_2_ + 1) − ln(n_1_ + 1)) of sites at which species occurred, and (**b**) occurrence change is calculated as ln O_2_ − ln O_1_. O_2_: number of sites at which species occurred in the second survey; O_1_: number of sites at which species occurred in the first survey. In the Wilcoxon Rank-Sum test, (**a**) *W* = 515, *p* = 0.003, and (**b**) *W* = 515, *p* = 0.003.

The changes in occurrence and abundance for the 87 taxa analyzed showed a significant positive correlation (Figure 4, Table S4). Changes in both abundance and occurrence were significantly influenced by the difficulty of identification (Figure 5). Specifically, difficult-to-identify taxa showed significantly greater increases than easy-to-identify taxa (Figure 5). For easy-to-identify taxa, the mean ± SD abundance change was 0.10 ± 0.63, whereas for difficult-to-identify taxa, it was 0.52 ± 0.82. Similarly, changes in occurrence were 0.18 ± 0.89 for easy-to-identify taxa and 0.88 ± 1.77 for difficult-to-identify taxa, indicating a substantial difference between groups. These results suggest that losses during identification were more pronounced in difficult-to-identify taxa than in the easy-to-identify taxa.

Table 2 compares the results of this study with those from previous studies that predicted changes in beetle abundance and occurrence under climate change scenarios. The fact that the primary impact of identifier bias was the underestimation of abundance in the first survey (Figure 3) may be reflected by observed increases in the second survey; decreases are more likely to indicate actual ecological changes. Therefore, to minimize the effects of the identifier, comparisons between predictions and observations were only made for taxa showing decreases in abundance and/or occurrence. Among these, 12 taxa showed changes consistent with predictions, whereas 3 taxa did not, yielding a statistically significant difference (*χ*^2^ = 5.4, *p* = 0.02). This suggests that observed decreases in abundance and occurrence are largely in line with climate change projections, supporting the predictive validity of previous modeling efforts.

## 4. Discussion

### 4.1. Identifier Bias

This study highlights the practical challenges of long-term beetle monitoring across numerous sites when assessing the impacts of climate change on beetle distribution and diversity. A comparison of surveys conducted 10 years apart at 273 forest sites in South Korea revealed that the number of recorded species more than doubled, despite having nearly identical locations, similar seasons, and consistent methods. The number of individuals also varied significantly, primarily depending on the ease of species identification. These discrepancies are attributed to different identifier expertise rather than to actual ecological changes. Furthermore, differences in abundance and occurrence between periods were also strongly affected by the identifier. In long-term insect monitoring, changes in investigators and identifiers are inevitable and present a serious practical issue. Although often overlooked, distortions of survey results by various identifiers can be substantial, as demonstrated in this study.

Identifier-related discrepancies likely arose primarily during the preparation of dried specimens prior to species identification. In the second survey, T.-S. Kwon, who handled both specimen preparation and identification, had relatively extensive experience with beetles. In contrast, in the first survey, specimen preparation was conducted by an individual (Female A) who had no prior experience with beetles. Limited ability to distinguish between species is considered a primary cause of the observed differences. It is well known that when individuals encounter unfamiliar organisms, they tend to focus more on similarities than on distinguishing differences. This cognitive tendency may explain some taxonomic oversights. For example, novice identifiers may fail to detect subtle morphological differences between species. The involvement of laypeople or parataxonomists with limited experience is common in ecological studies [35,36,37]. Although high correlations are often reported between lay and expert identification [38], the substantial bias observed here may represent an exceptional case due to individual limitations. Nevertheless, the potential for bias stemming from an identifier’s expertise warrants further investigation across broader taxonomic groups.

### 4.2. High Species Richness

Of the 1061 beetle species recorded across the two surveys, 399 were identified at the species level, accounting for 38% of the total. A global meta-analysis of Staphylinidae reported species-level identification rates of 40–100% in the Palearctic period, 90–100% in the Nearctic period, and 0–50% in the Neotropical period, reflecting strong regional variation in taxonomic research [39]. In South Korea, seven taxonomists identified 1455 beetle species at a species-level identification rate of 57% [29,30]; for beetles collected using pitfall traps, Malaise traps, and sweeping at seven Wando Arboretum sites, the rate was 70% [28]. Thus, the species-level identification rate in this study is somewhat lower than previously reported. This relatively low rate may reflect the fact that second-survey specimens were identified solely by T.-S. Kwon, who is not a trained taxonomist and uses the literature and reference collections as a guide. Of the 822 species recorded in the second survey, 503 species (61%) were newly detected and not included in the 1455 species reference collection prepared for this study. This reference collection comprises beetles collected primarily via pitfall traps from 485 forest sites across South Korea over the past 20 years [30]. The large number of newly recorded species that were not present in the existing reference collection suggests high beetle diversity in Korean forests. Notably, newly recorded species were significantly smaller in body size than the previously recorded species (existing species: 8.9 ± 7.0 mm; newly recorded species: 4.1 ± 3.5 mm; Wilcoxon rank sum test, *W* = 39062, *p* < 2.2 × 10^−16^). Many of the new records comprised small detritivorous or microbivorous species associated with litter decomposition (Table S6).

### 4.3. Projections of Abundance and Occurrence of Diverse Korean Beetles

When comparing predicted and observed changes in taxa that exhibited declines in abundance and distribution—assumed to be unaffected by identifier bias—the number of taxa for which predictions matched observed outcomes was significantly higher than the number of unmatched outcomes. This suggests that climate change is indeed influencing beetle populations. Unlike the commonly used SDMs, the ETI method makes predictions based on a simple rule: if the annual mean temperature of a region is lower than the species’ ETI, an increase in abundance and distribution is predicted; if the temperature is higher, a decrease is expected [15,16]. However, because both SDMs and ETI rely on correlations between species distributions and temperature, their underlying predictive principles are not fundamentally different. For rare or species-rich taxa, ETI can serve as a practical alternative to SDMs for qualitatively predicting whether abundance and distribution are likely to increase or decrease under climate change. While SDMs are widely used for quantitative predictions, accuracy is often limited by climate scenario uncertainty and certain environmental and ecological factors that interact, such as urbanization, vegetation changes, acid rain, interspecific competition, and predation [14]. Therefore, in many predictive studies, the most critical information is not the exact numerical forecast, but whether a species is expected to increase or decrease due to climate change.

Predictions for beetle abundance and distribution in South Korea were made for approximately 30 years into the future (2056–2065) [15,16,17]. Although temperature did not change during the survey period (2007–2019, 13 years), the long-term warming trend (an average increase of 0.025 °C per year) suggests that detectable effects may begin to appear [23]. Accordingly, we expected the predicted changes to emerge during the second survey period (2017–2019), ten years after the first survey; the results of this study align with those expectations. For some species, distribution and abundance change linearly with temperature increases, whereas others exhibit more complex, non-linear responses such as an initial increase followed by a decline [40]. Therefore, we anticipate that agreement between observed and predicted patterns will increase over time as warming progresses. The results of both quantitative and qualitative predictions suggest that, overall, more beetle taxa (both families and species) are expected to decline (78 taxa) than to increase (49 taxa) in response to rising temperatures [15,16]. The high number of beetle taxa predicted to decrease in South Korea may reflect a “peninsula effect,” which limits the influx of southern species into the region. South Korea has a greater number of northern species adapted to cold climates, while southern species adapted to warmer conditions are less common [41]. As temperatures rise, cold-adapted species are more vulnerable to local extinction, and the opportunity for southern species to migrate northward into the peninsula is restricted. The species-level ETI (or STI, Species Temperature Index) has been widely used to assess distributional responses to climate change [31]. However, limited research has applied ETI to higher taxonomic levels (e.g., the genus or family levels), despite its promising potential. Notably, ref. [42] found that a family-level ETI of Diptera (referred to as the Family Temperature Index) was strongly correlated with the mean temperatures of survey sites. This finding suggests that higher-level ETI metrics could be valuable tools for studying and predicting distributional shifts caused by climate change.

### 4.4. Family-Level Indicators for Climate Change

In climate change research, selecting appropriate indicators is crucial for detecting ecological responses to global warming. One of the primary objectives of indicator selection is to concentrate research efforts on a small number of species or taxa that are particularly sensitive to climate change, i.e., those with an abundance and distribution that is strongly correlated with temperature, so that the impacts can be detected more clearly and efficiently. Typically, indicators are chosen at the species level. However, higher taxonomic levels, such as genus, family, or even order, can also serve as effective indicators, particularly when they exhibit tolerances to narrow temperature ranges. For example, stoneflies and aphids are diverse and abundant in temperate regions but are markedly reduced or nearly absent in tropical areas [2]. This pattern reflects their evolutionary origin in cooler temperate climates [2,43]. As temperate zones become warmer and more subtropical, these taxa are expected to decrease. In this context, Cantharidae, a family found in large numbers in colder regions, may serve as a useful indicator of climate change [44]. The ETI of Cantharidae is relatively low, at 9.4 °C (10.2 ± 0.7 mean ± SD, based on 23 families; Table S3). Because Cantharidae are predatory, they may respond more directly to climate change compared to through host plant dynamics. Ten species of Cantharidae—including *Asiopodabrus circumangulatus*—were recorded in this study. However, all species were rare (with an occurrence frequency of 1–18) and thus excluded from species-level comparisons. Nonetheless, family-level patterns can still be informative. Although a decline in Cantharidae was predicted based on their low ETI [16], an increase was observed. Similarly, for Cerambycidae, a family that includes many northern-adapted species [45] and has a relatively low ETI (9.7 °C), a decline was predicted, yet an increase was detected. However, as noted earlier, these apparent increases may partly reflect the underestimation of the first survey due to identifier bias. Cerambycidae, in particular, includes many species that feed on deadwood, and their populations may increase in response to tree mortality caused by pests or strong winds [2]. Because temperature and environmental disturbances can exert opposing influences, predicting trends in Cerambycidae based solely on temperature is challenging. Moreover, although pitfall traps are not optimal for Cerambycidae, continued surveys using pitfall traps across a wide range of forest sites, such as in this study, are expected to reveal meaningful trends in their population dynamics over time.

Leiodidae, a family predicted to decline under climate change due to its low ETI [16], also represents a promising indicator of climate change. Leiodidae are commonly collected in pitfall traps and are detritivorous beetles that feed on vertebrate carcasses, excrement, and microorganisms such as fungi [46]. In South Korea, they are more frequently found in high-altitude mountainous areas (Table S5). The ETI for Leiodidae was 9.27 °C, which is the second-lowest ETI among the 23 families analyzed, after Melandryidae (9.19 °C). However, Melandryidae are not suitable indicators due to the substantial morphological diversity that complicates their identification. By contrast, families such as Scolytidae (ETI = 10.76 °C) and Nitidulidae (ETI = 11.02 °C), which have relatively high ETI values, are suitable indicators and are expected to increase in abundance and distribution with rising temperatures. As predicted, both families showed increases in this study. However, due to their small body size, some of the observed increases may have been influenced by identifier bias. As wood-feeding beetles, Scolytidae are also expected to respond to increasing environmental disturbances associated with climate change, such as forest fires and tree mortality caused by drought or strong winds, which may act synergistically with temperature to enhance their abundance. Nitidulidae, on the other hand, benefit from a diverse diet that is primarily detritivorous and includes mycophagy [47], allowing them to be less dependent on specific host plants. Carabidae, the most frequently and abundantly collected family in pitfall traps, were predicted to exhibit minimal changes in response to climate change [16] and, thus, were excluded from Table 2. However, in this study, both the abundance and occurrence of Carabidae declined (Table S3). Since Carabidae are relatively large and morphologically distinct, they are less susceptible to identification errors. Therefore, the observed decline is likely to reflect a genuine population decrease between the two survey periods. Staphylinidae, another family collected in large numbers alongside Carabidae, were predicted to decline but instead showed an increase in this study. As discussed previously, this apparent increase may not reflect a true population trend, since Staphylinidae are assumed to be the most affected by identifier-related biases due to their small size and unclear morphological characteristics.

### 4.5. Genus-Level Indicators for Climate Change

The genus *Synuchus* was the most abundant beetle taxon in South Korea during both survey periods, accounting for 25–26% of all individuals collected. Although 13 species of Synuchus have been reported in South Korea [48], distinguishing them based on external morphology alone is challenging. While species-level identification is difficult, identifying the genus itself is relatively straightforward. With a low ETI of 9.75 °C, Synuchus is expected to decline in response to climate warming. However, in this study, its abundance increased while its occurrence decreased, presenting conflicting trends. As *Synuchus* species are predatory and highly abundant, changes in their population dynamics and distribution due to climate change are likely to have cascading effects on arthropod communities. The genus *Eucarabus* comprises large-bodied carabid beetles typically found in high mountain regions and is similarly expected to decline under warming conditions due to its even lower ETI (9.1 °C). Historically, only one species, *Carabus sternbergi*, was known to inhabit South Korea [49], but this number has since increased to eight species [48]. Like *Synuchus*, species-level identification of *Eucarabus* is difficult and often requires an examination of genitalia. As *Eucarabus* is more strongly associated with colder environments than *Synuchus*, it is expected to be more sensitive to rising temperatures. Consistent with this expectation, *Eucarabus* showed declines in both abundance and occurrence during the second survey period.

### 4.6. Species-Level Indicators for Climate Change

Among the 19 beetle species analyzed at the species level, 7 were identified as promising indicators of climate change based on temperature sensitivity and ease of identification. Two of these seven indicator species, the large-bodied carabids *Coptolabrus jankowskii* and *Coptolabrus smaragdinus*, were both predicted to decline in response to climate change [15,17]. However, contrary to expectations, *C. smaragdinus* increased in abundance. Both species are predatory and endemic to East Asia, occurring in Russia, China, and Korea [27]. In contrast, *Chlaenius naeviger*, a medium-sized carabid beetle, was predicted to increase due to its relatively high ETI of 11.5 °C, but a decrease was observed in this study. *C. naeviger* is a habitat generalist, occupying a variety of environments including forests, farmlands, grasslands, shrublands, and riverbanks. Its geographic distribution includes Japan, China, and Taiwan [27].

Although the family Staphylinidae comprises numerous species with challenging identification, *Ocypus coreanus*, a species endemic to Eastern Asia, including Korea and the Russian Far East [50], is the largest staphylinid species in the country and is relatively easy to identify. This species is vulnerable to climate change; it inhabits mountainous areas in large numbers and exhibits a very low ETI of 8.06 °C (Table S4). Consistent with expectations, a significant decline was observed in *O. coreanus* populations, highlighting the need for conservation [17]. Another large staphylinid, *Agelosus weisei*, characterized by distinctive golden setae on its head and terminal abdomen, was also predicted to decline despite its relatively high ETI of 10.95 °C. Its abundance and distribution negatively correlate with rainfall [17], suggesting that increased precipitation associated with climate change may drive its decline more than temperature effects. Interestingly, however, declines in this species were predicted even when considering temperature alone [15].

*Nicrophorus quadripunctatus*, the most commonly collected species of Silphidae and a carrion feeder, was predicted to decline due to climate change: a trend that was confirmed by the significant decrease observed in this study. Given its close ecological association with vertebrates such as mammals, reptiles, and amphibians through its feeding habits, the decline of *N. quadripunctatus* may serve as an indicator of the effects of climate change on vertebrate populations. In contrast, *Onthophagus atripennis*, a dung beetle closely associated with vertebrates due to its reliance on vertebrate excrement, exhibited an increase, presenting conflicting results. However, this increase cannot exclude the potential influence of identifier bias. The excrement of wild boars, raccoons, and rodents is abundant in Korean forests and is presumed to serve as a food source for *O. atripennis*; however, no empirical studies have confirmed this. Among scarabid beetles, *O. atripennis* is the most frequently and abundantly collected species by pitfall traps and has a broad distribution across Korea as well as China and Japan [26]. Although mainly distributed in lowland areas, this species warrants monitoring for potential shifts in its altitudinal range in response to climate change; an upward movement is anticipated [17].

### 4.7. Evidence of Climate Warming and Difficulty in Entire Beetle Monitoring

Long-term monitoring across multiple sites is essential in order to understand the impacts of climate change on insect distribution. Due to the high species richness of beetles and the inherent difficulties in species-level identification, there are few examples of long-term monitoring studies covering the entire Coleoptera order across numerous survey sites. Consequently, predicted changes in abundance and distribution have generally been limited to a small subset of relatively common taxa that are amenable to robust statistical analysis. In this study, we compared beetle communities surveyed using consistent methods during two periods (2007–2009 and 2017–2019) at 273 forest sites. We found that the influence of identifiers on observed changes in species richness, abundance, and occurrence was substantial. This bias arose primarily from differences in the taxonomic experience of the identifiers and was especially pronounced in taxa that are morphologically difficult to identify. Since identifier bias tended to cause an underestimation of abundance and occurrence in the first survey, increases in the second survey may partly reflect this bias, and decreases are more likely to represent true ecological changes. In other words, declines in abundance and occurrence are relatively less affected by identifier bias, though some underestimation may remain. When comparing predicted and observed changes, we restricted analysis to taxa exhibiting decreases in abundance and occurrence, for which identifier bias is less problematic. In this subset, agreement with predictions significantly outnumbered disagreements, providing empirical support that climate change-driven shifts in beetle populations are occurring in nature. Considering ease of identification, abundance, and sensitivity to climate change, fourteen taxa, including five families, two genera, and eight species, were identified as promising indicators for monitoring the impacts of climate change. However, this study was unable to reliably detect changes in richness, abundance, and occurrence across the entire Coleoptera order due to the unexpectedly strong influence of identifier bias. Data encompassing the full Coleoptera order would lead to greater understanding regarding biodiversity changes and ecosystem responses rather than data limited to individual families; thus, it is critical to develop methods to minimize identifier-related biases and enhance the accuracy, reliability, and consistency of taxonomic identifications. Such advances will be essential for effectively utilizing broad-scale Coleoptera data to assess the ecological impacts of climate change.

## 5. Conclusions

This study provides empirical evidence that beetle communities in South Korean forests are responding to climate change. This is reflected by the shifts in species richness, abundance, and occurrence observed over a ten-year period. However, we also found that these changes are significantly influenced by identifier bias, particularly in taxa that are small or morphologically similar. Increases in species richness and abundance were often associated with improved identification accuracy in the second survey, while decreases were more likely to represent genuine ecological shifts. When comparing observed decreases to prior predictions based on species distribution models and ecological temperature indices, a strong concordance was found, reinforcing the predictive validity of climate-driven models. Based on ease of identification, abundance, and temperature sensitivity, we propose fourteen indicator taxa—including five families, two genera, and seven species—for long-term climate change monitoring. These findings highlight both the challenges and opportunities presented by biodiversity monitoring, as well as the importance of consistent methodologies and expert taxonomic oversight to improve the reliability of long-term ecological assessments.

## Figures and Tables

**Table 1:** Different identifiers in the first (2007–2009) and second (2017–2019) surveys. A and B: anonymous trained laywomen who counted and mounted beetles. T.-S. Kwon: insect ecologist, C.M. Lee: Japanese carabid ecologist, S.-S. Kim: Lepidoptera taxonomist, J.K. Jung: Korean carabid ecologist, S.K. Lee: staphylinid taxonomist, S.W. Park: curculionid taxonomist with extensive experience in the identification of various beetle taxa.

Identification	First Survey	Second Survey
Counting number of beetles (order level)	A	B
Dry mounting and counting number of species	A	T.-S. Kwon
First identification		
(identification by books)	C.M. Lee (Carabidae) S.-S. Kim (Other)	T.-S. Kwon
Second identification		
(identification by specialist)	J.K. Jung (Carabidae) S.K. Lee (Staphylinidae) S.W. Park (Other)	
(identification by comparison with reference specimen)		T.-S. Kwon

**Table 2:** Changes in abundance and occurrence of common taxa predicted by the previous studies [15,16]. Abundance change = mean of site abundance change (ln (second survey abundance + 1) − ln (first survey abundance + 1)); occurrence change = ln (number of sites in which species occurred in the second survey) − ln (number of sites in which species occurred in the first survey). First survey: 2007–2009; second survey: 2017–2019. Prediction of future abundance (2056–2065) was conducted using the SDM or ETI method. Taxa with thick letters are easily identified climate change indicators, the abundance and occurrence of which are highly dependent on climate warming in South Korea. ETI: abundance-weighted average of annual mean temperature at the sites where the species occurred (see text for calculation).

Taxa Level	Taxa Name	Change		Prediction	Method	Coincidence		ETI Identification
		Abundance	Occurrence					
**Family**	**Cantharidae**	**0.89**	**2.08**	**Decrease**	**ETI**	**No**	**9.38**	**Easy**
**Family**	**Cerambycidae**	**0.28**	**0.48**	**Decrease**	**ETI**	**No**	**9.70**	**Easy**
Family	Curculionidae	0.27	0.10	Decrease	SDM	No	9.93	Easy
Family	Elateridae	0.33	0.47	Decrease	SDM	No	10.37	Easy
Family	Histeridae	0.15	0.19	Decrease	ETI	No	10.44	Easy
Family	Hydrophilidae	−0.21	−0.39	Decrease	SDM	Yes	10.04	Difficult
**Family**	**Leiodidae**	**1.01**	**1.08**	**Decrease**	**ETI**	**Yes**	**9.27**	**Easy**
Family	Melandryidae	0.73	2.60	Decrease	ETI	No	9.19	Easy
Family	Melonlonthidae	−0.47	−0.46	Decrease	SDM	Yes	10.01	Easy
Family	Mordellidae	0.96	2.07	Increase	ETI	Yes	11.47	Easy
**Family**	**Nitidulidae**	**1.14**	**0.98**	**Increase**	**ETI**	**Yes**	**11.03**	**Easy**
Family	Scarabaeidae	0.43	0.04	Increase	SDM	Yes	11.44	Easy
**Family**	**Scolytidae**	**0.74**	**1.57**	**Increase**	**ETI**	**Yes**	**10.76**	**Easy**
Family	Silphidae	−0.23	−0.17	Decrease	SDM	Yes	9.67	Easy
Family	Staphylinidae	0.94	0.11	Decrease	SDM	No	10.26	Easy
Family	Tenebrionidae	0.66	0.34	Decrease	SDM	No	10.55	Easy
Genus	Atheta	1.56	1.30	Decrease	ETI	No	9.96	Difficult
**Genus**	**Eucarabus**	**−0.24**	**−0.49**	**Decrease**	**SDM**	**Yes**	**9.09**	**Easy**
Genus	Misolampidius	0.35	0.25	Decrease	SDM	No	9.61	Easy
**Genus**	**Synuchus**	**0.10**	**−0.06**	**Decrease**	**ETI**		**9.75**	**Easy**
**Species**	***Agelosus weisei***	**−0.14**	**−0.20**	**Decrease**	**SDM**	**Yes**	**10.95**	**Easy**
Species	*Anaedius mroczkowskii*	−0.07	0.12	Decrease	SDM		10.88	Easy
Species	*Anomotarsus stigmula*	0.68	2.20	Increase	ETI	Yes	12.16	Difficult
Species	*Aulacosypus parvulus*	−0.30	−0.16	Increase	SDM	No	11.03	Difficult
Species	*Aulonocarabus semiopacus*	0.49	0.46	Decrease	ETI	No	7.90	Difficult
**Species**	***Chlaenius naeviger***	**−0.53**	**−0.38**	**Increase**	**SDM**	**No**	**11.50**	**Easy**
**Species**	***Chromogeotrupes auratus***	**0.67**	**1.39**	**Decrease**	**ETI**	**No**	**9.48**	**Easy**
**Species**	***Coptolabrus jankowskii***	**−0.17**	**−0.23**	**Decrease**	**SDM**	**Yes**	**10.91**	**Easy**
**Species**	***Coptolabrus smaragdinus***	**0.44**	**0.78**	**Decrease**	**ETI**	**No**	**10.01**	**Easy**
Species	*Hylobitelus haroldi*	−0.44	−0.69	Decrease	SDM	Yes	9.55	Easy
**Species**	***Nicrophorus quadripunctatus***	**−0.41**	**−0.34**	**Decrease**	**SDM**	**Yes**	**9.42**	**Easy**
**Species**	***Ocypus coreanus***	**−0.63**	**−0.82**	**Decrease**	**SDM**	**Yes**	**8.06**	**Easy**
**Species**	***Onthophagus atripennis***	**0.58**	**0.13**	**Increase**	**SDM**	**Yes**	**11.63**	**Easy**
Species	*Planetes puncticeps*	−0.42	−0.43	Increase	SDM	No	12.06	Easy
Species	*Platydracus brevicornis*	−0.96	−0.64	Decrease	SDM	Yes	10.73	Easy
Species	*Pristosia vigil*	0.37	0.23	Decrease	ETI	No	7.88	Difficult
Species	*Pterostichus (Koreonialoe)* spp.	−0.57	−0.62	Decrease	ETI	Yes	8.12	Easy
Species	*Pterostichus audax*	1.30	1.04	Decrease	ETI	No	8.91	Difficult
Species	*Pterostichus orientalis*	−1.21	−1.07	Decrease	ETI	Yes	8.58	Easy

## Data Availability

The data presented in this study are available on request from the corresponding author due to ethical reasons.

## Supplementary Materials

The following supporting information can be downloaded at: https://www.mdpi.com/article/10.3390/insects17020156/s1, Table S1: Annual change of climate; Table S2: Data of sampling sites; Table S3: Data of body size of occurred species; Table S4: Data of change of abundance and occurrence, and ETI; Table S5: Data of abundance of occurred species; Table S6: Data of averaged abundance and occurrence of occurred species.

## Abbreviations

The following abbreviations are used in this manuscript:

ETI	Ecological Temperature Index
SDMs	Species Distribution Models
